# *APOL1* genetic variants, chronic kidney diseases and hypertension in mixed ancestry South Africans

**DOI:** 10.1186/s12863-015-0228-6

**Published:** 2015-06-26

**Authors:** Tandi E Matsha, Andre P Kengne, Katya L Masconi, Yandiswa Y Yako, Rajiv T Erasmus

**Affiliations:** Department of Biomedical Sciences, Faculty of Health and Wellness Science, Cape Peninsula University of Technology, PO Box 1906, Bellville, Cape Town 7530 South Africa; Non-Communicable Diseases Research Unit, South African Medical Research Council & University of Cape Town, Cape Town, South Africa; Department of Surgery, Faculty of Health Sciences, University of Witwatersrand, Johannesburg, South Africa; Division of Chemical Pathology, Faculty of Health Sciences, National Health Laboratory Service (NHLS) and University of Stellenbosch, Cape Town, South Africa

**Keywords:** Africa, APOL1 polymorphisms, Chronic kidney diseases, Blood pressure, MDRD, CKD-EPI

## Abstract

**Background:**

The frequencies of apolipoprotein L1 (*APOL1)* variants and their associations with chronic kidney disease (CKD) vary substantially in populations from Africa. Moreover, available studies have used very small sample sizes to provide reliable estimates of the frequencies of these variants in the general population. We determined the frequency of the two *APOL1* risk alleles (G1 and G2) and investigated their association with renal traits in a relatively large sample of mixed-ancestry South Africans. *APOL1* risk variants (G1: rs60910145 and rs73885319; G2: rs71785313) were genotyped in 859 African mixed ancestry individuals using allele-specific TaqMan technology. Glomerular filtration rate (eGFR) was estimated using the Modification of Diet in Renal Disease (MDRD) and Chronic Kidney Disease Epidemiology Collaboration (CKD-EPI) equations.

**Results:**

The frequencies of rs73885319, rs60910145 and rs71785313 risk alleles were respectively, 3.6 %, 3.4 %, and 5.8 %, resulting in a 1.01 % frequency of the *APOL1* two-risk allele (G1:G1 or G1:G2 or G2:G2). The presence of the two-risk allele increased serum creatinine with a corresponding reduction in eGFR (either MDRD or CKD-EPI based). In dominant and log-additive genetic models, significant associations were found between rs71785313 and systolic blood pressure (both p ≤ 0.025), with a significant statistical interaction by diabetes status, p = 0.022, reflecting a negative non-significant effect in nondiabetics and a positive effect in diabetics.

**Conclusions:**

Although the *APOL1* variants are not common in the mixed ancestry population of South Africa, the study does provide an indication that *APOL1* variants may play a role in conferring an increased risk for renal and cardiovascular risk in this population.

## Background

In 2008, through admixture-mapping linkage-disequilibrium genome scan, two landmark studies identified a risk locus on chromosome 22q12.3 which explained the increased burden of nondiabetic end­stage renal disease (ESRD) and focal segmental glomerulosclerosis (FSGS) in individuals of recent African ancestry [1, 2]. These studies provided evidence that genetic variation on the myosin­9 gene (*MYH9*) conferred most or nearly all of the increased risk for nondiabetic kidney disease in African Americans [1, 2]. However, subsequent reanalysis of chromosome 22q12.3 region utilizing the 1000 Genome Project, identified genetic variants in the apolipoprotein L1 gene *(APOL1)* which extended beyond *MYH9* [3, 4]. These are located 14kbp downstream from the 3’end of *MYH9* and the strongest significant association with ESRD was found in a 10-kb region in the last exon of *APOL1* [3, 4]. Of the single nucleotide polymorphisms (SNPs) identified, two were nonsynonymous (rs73885139 and rs60910145) designated G1, and one a 6-bp deletion (rs71785313) termed G2. The two missense variants are in almost absolute linkage disequilibrium while the G2 is in complete negative linkage disequilibrium with G1.

The *APOL1* is known for its lytic effects on trypanosomes, which cause sleeping sickness in humans [5]. However, one of the Tryanosoma species (*T. brucei rhodesiense*) overcomes the lytic effects of *APOL1* by expressing a serum resistance-associated protein (SRA) [6]. The presence of G1 and G2 polymorphisms in the SRA binding domain are believed to restore the *APOL1* ability to kill *T. brucei rhodesiense* [7], hence the positive selection of the variants in endemic regions, particularly in sub-Saharan Africa. Variation in frequency is substantial within Africa; and therefore the contribution to chronic kidney disease (CKD) is likely to vary [4, 7]. However, at this time the geographic variation in *APOL1* association with CKD is unknown, since it has not yet been tested. Therefore, herein, we sought to determine the frequency of *APOL1* variants and their association with CKD traits in a South African population with an African ancestry, high prevalence of CKD and poor cardiovascular risk profile.

## Results

Two hundred and thirty nine (27.8 %) participants had diabetes and their general characteristics are summarized in Table 1. The overall mean age was 53.1 years, with significant differences between those with and without diabetes (51.0 vs. 58.7 years, p < 0.0001). The eGFR was significantly lower in individuals with diabetes compared to those without diabetes as well as in women vs. men (all p < 0.0001), whilst systolic blood pressure and diastolic blood pressure were significantly elevated in men (both p ≤ 0.016).

**Table 1:** Baseline characteristics by diabetes status and gender

Characteristics	No diabetes	Diabetes	p-value	Men	Women	p-value	Overall
N	620	239		195	664		859
Gender, men n (%)	140 (22.6)	55 (23.0)	0.892	195 (100)	-	NA	195 (22.7)
Mean age, years (SD)	51.0 (13.9)	58.7 (12.9)	<0.0001	54.5 (14.6)	52.7 (13.9)	0.140	53.1 (14.1)
Mean BMI, kg/m^2^ (SD)	29.4 (7.2)	31.7 (7.4)	<0.0001	26.4 (6.4)	31.1 (7.2)	<0.0001	30.0 (7.3)
Mean WHR (SD)	0.87 (0.10)	0.92 (0.09)	<0.0001	0.93 (0.08)	0.87 (0.10)	<0.0001	0.88 (0.10)
Mean SBP, mmHg (SD)	121 (19)	130 (23)	<0.0001	127 (19)	123 (21)	0.016	124 (21)
Mean DBP, mmHg (SD)	75 (12)	76 (13)	0.073	77 (12)	76 (13)	0.006	75 (12)
Hypertension, yes (%)	336 (54.2)	156 (65.3)	0.003	94 (48.2)	398 (59.9)	0.004	492 (57.3)
Mean HbA1c, % (SD)	5.7 (0.4)	7.8 (2.1)	<0.0001	6.3 (1.7)	6.3 (1.4)	0.901	6.3 (1.5)
Mean FBG, mmol/l (SD)	5.2 (0.7)	9.8 (4.3)	<0.0001	6.4 (3.5)	6.4 (3.0)	0.928	6.4 (3.1)
Mean 2-h glucose, mmol/l (SD)	6.5 (1.6)	13.3 (5.0)	<0.0001	6.9 (3.0)	7.8 (3.7)	0.004	7.6 (3.5)
Median urine creatinine, mmol/l (25^th^-75^th^ percentiles)	8.1 [5.3-12.2]	7.0 [4.3-11.3	0.045	10.3 [5.9-14.3]	7.3 [5.0-11.2]	<0.0001	7.9 [5.2-11.9]
Median urine Microalbumin, mg/l (25^th^-75^th^ percentiles)	3.7 [3.0-9.5]	7.6 [3.0-28.9]	<0.0001	5.7 [3.0-14.9]	4.1 [3.0-10.4]	0.093	4.4 [3.0-11.3]
Median ACR, mg/mmol (25^th^-75^th^ percentiles)	0.62 [0.37-1.25]	1.06 [0.59-3.27]	<0.0001	0.67 [0.32-1.79]	0.77 [0.45-1.54]	0.123	0.75 [0.41-1.57]
Median serum creatinine,μmol/l (25^th^-75^th^ percentiles)	79 [69-90]	82 [70-97]	0.016	92 [82-102]	77 [67-88]	<0.0001	80 [69-92]
Median eGFR (MDRD), ml/min (SD)	87.8 [73.7-101.9]	79.6 [66.6-96.3]	<0.0001	91.6 [77.5-105.8]	84.0 [69.9-98.9]	0.0001	85.8 [71.1-100.7
eGFR (MDRD) <60, n (%)	43 (6.9)	36 (15.1)	0.0002	13 (6.7)	66 (9.9)	0.164	79 (9.2)
Median eGFR (CKD-EPI), ml/min (SD)	94.9 [77.5-110.1]	82.8 [67.8-103.1]	<0.0001	96.5 [78.2-111.6]	90.0 [71.9-107.5]	0.033	91.2 [72.8-108.8]
eGFR (CKD-EPI) <60, n (%)	39 (6.3)	34 (14.2)	0.0002	14 (7.2)	59 (8.9)	0.453	73 (8.5)

ACR, urinary albumin/creatinine ratio; CKD-EPI, Chronic Kidney Disease Epidemiology Collaboration; eGFR, estimated glomerular filtration rate; MDRD, Modification of Diet in Renal Disease; SD, standard deviation

The frequency distributions, both genotype and allele, did not differ significantly according to gender and diabetes status. Deletion of the sequence TTATTA of rs71785313 was borderline more frequent in women than in men (6.3 % vs. 3.8 %, p = 0.065), (Table 2). The concomitance of two-risk alleles was observed in 9 individuals (1.01 %) whilst 143 (16.6 %) had one-risk allele (Table 3). In participants with two-risk alleles, serum creatinine was elevated with a corresponding reduction of eGFR (either MDRD or CKD-EPI based) than in those with only one-risk allele or none, but differences did not reach statistical significance. Furthermore, these were still more likely to have higher prevalence of hypertension (Table 3).

**Table 2:** Genotype distributions, minor allele frequencies, and unadjusted p-values for comparing genotype distributions according to diabetes status and gender

	No diabetes	Diabetes	p-value	Men	Women	p-value	Overall
N	620	239		195	664		859
**rs60910145**							
T/T, n (%)	582 (93.9)	220 (92.0)	0.355	179 (91.8)	623 (93.8)	0.380	802 (93.4)
T/G, n (%)	36 (5.8)	19 (7.9)		16 (8.2)	39 (5.9)		55 (6.4)
G/G, n (%)	2 (0.3)	0 (0)		0 (0)	2 (0.3)		2 (0.2)
G, n (%)	40 (3.2)	19 (4.0)	0.446	16 (4.1)	43 (3.2)	0.410	59 (3.4)
HWE (p-value)	0.127	>0.999		>0.999	0.145		0.263
**rs73885319**							
A/A, n (%)	580 (93.5)	219 (91.6)	0.346	179 (91.8)	620 (93.4)	0.492	799 (93.0)
A/G, n (%)	38 (6.1)	20 (8.4)		16 (8.2)	42 (6.3)		58 (6.7)
G/G, n (%)	2 (0.3)	0 (0)		0 (0)	2 (0.3)		2 (0.2)
G, n (%)	42 (3.4)	20 (4.2)	0.427	16 (4.1)	46 (3.5)	0.554	62 (3.6)
HWE (p-value)	0.150	>0.999		>0.999	0.181		0.302
**rs71785313**							
TTATTA / TTATTA, n (%)	555 (89.5)	210 (87.9)	0.704	181 (92.8)	584 (87.9)	0.154	765 (89.1)
TTATTA /Del, n (%)	62 (10.0)	27 (11.3)		13 (6.7)	76 (11.4)		89 (10.4)
Del/Del, n (%)	3 (0.5)	2 (0.8)		1 (0.5)	4 (0.6)		5 (0.6)
Del, n (%)	68 (11.0)	31 (6.5)	0.424	15 (3.8)	84 (6.3)	0.065	99 (5.8)
HWE (p-value)	0.420	0.253		0.244	0.324		0.195

HWE, Hardy-Weinberg Equilibrium (HWE p-values are from exact tests**)**

**Table 3:** Baseline characteristics by allele combination

Characteristics	0 risk allele	One risk allele	Two risk alleles	P-value*	p-value**
N	707	143	9		
Gender, men n (%)	166 (23.5)	27 (18.8)	2 (22.2)	0.488	0.972
Mean age, years (SD)	53.2 (14.0)	52.7 (10.0)	54.3 (10.2)	0.902	0.797
Mean BMI, kg/m^2^ (SD)	30.0 (7.5)	30.0 (6.7)	32.0 (6.9)	0.722	0.420
Mean WHR (SD)	0.88 (0.10)	0.89 (0.08)	0.92 (0.11)	0.484	0.266
Mean SBP, mmHg (SD)	123 (20)	127 (23)	121 (12)	0.113	0.737
Mean DBP, mmHg (SD)	75 (12)	77 (13)	75 (12)	0.117	0.884
Hypertension, yes (%)	466 (65.9)	98 (68.5)	8 (88.9)	0.301	0.118
Mean HbA1c, % (SD)	6.2 (1.4)	6.5 (1.7)	6.6 (1.5)	0.154	0.586
Mean FBG, mmol/l (SD)	6.3 (3.0)	6.9 (3.6)	7.0 (3.3)	0.140	0.599
Mean 2-h glucose, mmol/l (SD)	7.4 (3.2)	8.2 (4.7)	9.0 (4.2)	0.040	0.264
Median urine creatinine, mmol/l (25^th^-75^th^ percentiles)	7.9 [5.2-11.8]	7.9 [4.5-12.1]	5.6 [5.5-8.8]	0.664	0.466
Median urine Microalbumin, mg/l (25^th^-75^th^ percentiles)	4.4 [3.0-11.4]	4.3 [3.0-11.7]	3.0 [3.0-7.3]	0.537	0.293
Median ACR, mg/mmol (25^th^-75^th^ percentiles)	0.75 [0.41-1.61]	0.77 [0.41-1.50]	0.54 [0.34-1.06]	0.876	0.541
Median serum creatinine,μmol/l (25^th^-75^th^ percentiles)	80 [69-92]	79 [69-90]	85 [82-92]	0.254	0.244
Median eGFR (MDRD), ml/min (SD)	85.9 [70.8-102.2]	84.2 [73.9-99.1]	76.6 [73.6-81.0]	0.258	0.147
eGFR (MDRD) <60, n (%)	67 (9.5)	11 (7.7)	1 (11.1)	0.781	0.846
Median eGFR (CKD-EPI), ml/min (SD)	91.8 [72.0-109.9]	91.2 [75.2-107.9]	82.2 [77.1-85.2]	0.275	0.160
eGFR (CKD-EPI) <60, n (%)	60 (8.5)	12 (8.4)	1 (11.1)	0.960	0.786

ACR, urinary albumin/creatinine ratio; CKD-EPI, Chronic Kidney Disease Epidemiology Collaboration; eGFR, estimated glomerular filtration rate; MDRD, Modification of Diet in Renal Disease; SD, standard deviation

*p-values from chi square, ANOVA and Kruskal-Wallis tests

**p-values from recessive genetic models

In a recessive model adjusted for age, sex, diabetes status and hypertension, the G1 risk alleles showed a borderline association with prevalent CKD (CKD-EPI), p = 0.047 (Table 4). On the other hand, in generalized linear and logistics regression models (dominant and log-additive genetic models) adjusted for age, sex, diabetes status and hypertension, none of the genotypes was associated with serum creatinine, urinary albumin/creatinine ratio or prevalent CKD (Table 5 and 6). These genetic models revealed an association between G2 (rs71785313) and systolic blood pressure (both p ≤ 0.025), with a significant statistical interaction by diabetes status, p = 0.025, reflecting a negative non-significant effect in nondiabetics and a positive effect in diabetics.

**Table 4:** Generalized linear and logistic regression models showing the effects of genes on kidney functions and other continuous predictors (recessive model)

		Overall	
Allele	Phenotype	Effects size (95 % CI)	P
**rs60910145 G/G**	Serum creatinine (log_e_[μmol/l])	0.192 (-0.094 to 0.478)	0.289
	eGFR (MDRD) [ml/min]	−18.54 (-48.59 to 11.51)	0.227
	eGFR (CKD-EPI) [ml/min]	−18.90 (-43.76 to 5.96)	0.136
	Prevalent CKD (MDRD)	23.47 (0.92 to 599.29)	0.074
	Prevalent CKD (CKD-EPI)	42.72 (1.22 to ∞)	0.047
	Albumin/creatinine ratio (log_e_[mg/mmol])	−1.02 (-2.62 to 0.57)	0.210
	Systolic blood pressure (mmHg)	−0.36 (-27.67 to 26.74)	0.979
**rs73885319 G/G**	Serum creatinine (log_e_[μmol/l])	0.191 (-0.094 to 0.478)	0.189
	eGFR (MDRD) [ml/min]	−18.54 (-48.59 to 11.51)	0.227
	eGFR (CKD-EPI) [ml/min]	−18.90 (-43.76 to 5.96)	0.136
	Prevalent CKD (MDRD)	23.47 (0.92 to 599.29)	0.074
	Prevalent CKD (CKD-EPI)	42.72 (1.22 to ∞)	0.047
	Albumin/creatinine ratio (log_e_[mg/mmol])	−1.02 (-2.62 to 0.57)	0.210
	Systolic blood pressure (mmHg)	−0.36 (-27.67 to 26.94)	0.979
**rs71785313 Del/Del**	Serum creatinine (log_e_[μmol/l])	0.038 (-0.144 to 0.219)	0.684
	eGFR (MDRD) [ml/min]	−7.16 (-26.19 to 11.87)	0.461
	eGFR (CKD-EPI) [ml/min]	−5.99 (-21.74 to 9.76)	0.456
	Prevalent CKD (MDRD)	0.0	-
	Prevalent CKD (CKD-EPI)	0.0	-
	Albumin/creatinine ratio (log_e_[mg/mmol])	−0.123 (-1.134 to 0.888)	0.811
	Systolic blood pressure (mmHg)	−6.27 (-23.55 to 11.02)	0.478

Models are adjusted for age, sex, diabetes status and hypertension

Effect estimates are mean difference and 95 % confidence intervals for quantitative traits and odds ratio and 95 % confidence intervals for qualitative traits

CKD-EPI, Chronic Kidney Disease Epidemiology Collaboration; eGFR, estimated glomerular filtration rate; MDRD, Modification of Diet in Renal Disease

**Table 5:** Generalized linear and logistic regression models showing the effects of genes on kidney functions and other continuous predictors (dominant model)

		Overall		Interaction p for the gene and		
Allele	Phenotype	Effects size (95 % CI)	P	Sex	diabetes	hypertension
**rs60910145 T/G-G/G**	Serum creatinine (log_e_[μmol/l])	−0.029 (-0.084 to 0.026)	0.307	0.658	0.176	0.158
	eGFR (MDRD) [ml/min]	2.09 (-3.73 to 7.91)	0.482	0.444	0.199	0.101
	eGFR (CKD-EPI) [ml/min]	3.24 (-1.57 to 8.06)	0.187	0.768	0.143	0.103
	Prevalent CKD (MDRD)	0.59 (0.18 to 1.89)	0.350	0.677	0.081	0.410
	Prevalent CKD (CKD-EPI)	0.84 (0.27 to 2.65)	0.767	0.244	0.118	0.456
	Albumin/creatinine ratio (log_e_[mg/mmol])	−0.154 (-0.502 to 0.193)	0.384	0.331	0.947	0.525
	Systolic blood pressure (mmHg)	−0.02 (-5.31 to 5.27)	0.993	0.839	0.536	0.963
**rs73885319 A/G-G/G**	Serum creatinine (log_e_[μmol/l])	−0.026 (-0.080 to 0.028)	0.341	0.612	0.260	0.273
	eGFR (MDRD) [ml/min]	1.75 (-3.93 to 7.44)	0.546	0.402	0.283	0.181
	eGFR (CKD-EPI) [ml/min]	2.96 (-1.74 to 7.66)	0.217	0.708	0.233	0.264
	Prevalent CKD (MDRD)	0.56 (0.18 to 1.79)	0.307	0.654	0.086	0.411
	Prevalent CKD (CKD-EPI)	0.81 (0.26 to 2.54)	0.720	0.233	0.123	0.455
	Albumin/creatinine ratio (log_e_[mg/mmol])	−0.096 (-0.436 to 0.245)	0.583	0.240	0.883	0.411
	Systolic blood pressure (mmHg)	−0.020 (-5.18 to 5.14)	0.994	0.834	0.585	0.837
**rs71785313** TTATTA /Del-Del/Del	Serum creatinine (log_e_[μmol/l])	0.020 (-0.024 to 0.064)	0.382	0.549	0.801	0.891
	eGFR (MDRD) [ml/min]	−2.35 (-6.99 to 2.30)	0.323	0.479	0.865	0.893
	eGFR (CKD-EPI) [ml/min]	−3.03 (-6.88 to 0.81)	0.123	0.478	0.815	0.988
	Prevalent CKD (MDRD)	0.91 (0.38 to 2.14)	0.823	0.934	0.639	0.782
	Prevalent CKD (CKD-EPI)	1.07 (0.43 to 2.66)	0.890	0.971	0.487	0.622
	Albumin/creatinine ratio (log_e_[mg/mmol])	0.050 (-0.214 to 0.314)	0.710	0.587	0.854	0.889
	Systolic blood pressure (mmHg)	5.60 (1.39 to 9.80)	0.009	0.892	0.025	0.593

Models are adjusted for age, sex, diabetes status and hypertension

Effect estimates are mean difference and 95 % confidence intervals for quantitative traits and odds ratio and 95 % confidence intervals for qualitative traits

CKD-EPI, Chronic Kidney Disease Epidemiology Collaboration; eGFR, estimated glomerular filtration rate; MDRD, Modification of Diet in Renal Disease

**Table 6:** Generalized linear and logistic regression models showing the effects of genes on kidney functions and other continuous predictors (additive model)

		Overall	
Allele	Phenotype	Effects size (95%CI)	P
**rs60910145 G**	Serum creatinine (log_e_[μmol/l])	−0.020 (-0.072 to 0.033)	0.466
	eGFR (MDRD) [ml/min]	1.26 (-4.27 to 6.79)	0.656
	eGFR (CKD-EPI) [ml/min]	2.28 (-2.29 to 6.86)	0.328
	Prevalent CKD (MDRD)	0.80 (0.28 to 2.27)	0.665
	Prevalent CKD (CKD-EPI)	1.12 (0.39 to 3.16)	0.836
	Albumin/creatinine ratio (log_e_[mg/mmol])	−0.178 (-0.504 to 0.147)	0.283
	Systolic blood pressure (mmHg)	−0.03 (-5.06 to 4.99)	0.989
**rs73885319 G**	Serum creatinine (log_e_[μmol/l])	−0.018 (-0.069 to 0.034)	0.503
	eGFR (MDRD) [ml/min]	0.99 (-4.42 to 6.40)	0.720
	eGFR (CKD-EPI) [ml/min]	2.07 (-2.40 to 6.55)	0.364
	Prevalent CKD (MDRD)	0.76 (0.27 to 2.16)	0.601
	Prevalent CKD (CKD-EPI)	1.08 (0.38 to 3.03)	0.887
	Albumin/creatinine ratio (log_e_[mg/mmol])	−0.126 (-0.446 to 0.195)	0.442
	Systolic blood pressure (mmHg)	−0.03 (-4.95 to 4.89)	0.990
**rs71785313 Del**	Serum creatinine (log_e_[μmol/l])	0.019 (-0.022 to 0.060)	0.367
	eGFR (MDRD) [ml/min]	−2.38 (-6.68 to 1.93)	0.323
	eGFR (CKD-EPI) [ml/min]	−2.91 (-6.46 to 0.65)	0.110
	Prevalent CKD (MDRD)	0.86 (0.39 8to 1.93)	0.712
	Prevalent CKD (CKD-EPI)	1.00 (0.42 to 2.34)	0.993
	Albumin/creatinine ratio (log_e_[mg/mmol])	0.035 (-0.207 to 0.277)	0.777
	Systolic blood pressure (mmHg)	4.48 (0.58 to 8.38)	0.025

Models are adjusted for age, sex, diabetes status and hypertension

Effect estimates are mean difference and 95 % confidence intervals for quantitative traits and odds ratio and 95 % confidence intervals for qualitative traits. CKD-EPI, Chronic Kidney Disease Epidemiology Collaboration; eGFR, estimated glomerular filtration rate; MDRD, Modification of Diet in Renal Disease

## Discussion

This study aimed to determine the frequency of the two *APOL1* risk alleles (G1 and G2) and their association with renal traits in a general South African mixed ancestry population. The findings from this study show that 16.6 % of the participants carried at least one *APOL1* risk allele. The G1 risk alleles, rs73885319 and rs60910145 were observed respectively in, 3.6 %, 3.4 % of individuals whilst G2 risk allele carriers were 5.8 %, resulting in a 1.01 % frequency of the *APOL1* two-risk allele (G1:G1 or G1:G2 or G2:G2). The presence of two-risk alleles decreased eGFR and the G1 risk alleles showed a borderline association with prevalent CKD (CKD-EPI), p = 0.047. On one hand, the G2 leaned towards an association with systolic blood pressure (p ≤ 0.025), with a significant statistical interaction by diabetes status, p = 0.025 assuming either dominant or log-additive. Our findings in the context of a small sample could also reflect the inadequate statistical power for uncovering some significant associations.

The strong association between *APOL1* and non-diabetic kidney diseases has been replicated in several studies [8–11] since the initial observations reported in African Americans with hypertensive kidney disease and FSGS [3, 4]. This risk is mostly conferred by the presence of two copies of the risk alleles, that is, homozygous or compound heterozygous compared to no or one *APOL1* risk variant [3, 4]. In our study, the frequency of the two-risk allele was much lower than that reported in African Americans (13 %) [12], and it was borderline associated with CKD or its markers. Furthermore, carriers of the two-risk allele were more likely to have hypertension. Our findings may be attributed to the non-discriminatory nature of our study in which diabetic and non-diabetic kidney diseases were not analyzed separately and would have resulted in an even smaller number of CKD cases. Nevertheless, our study does provide an indication that *APOL1* variants may play a role in conferring a poor renal disease and cardiovascular risk profile in this population. In linear genetic regression models, the G2 risk allele was significantly associated with systolic blood pressure. Emerging data point to an expanding role of *APOL1* genetic aberrations implying that they are not limited to kidney diseases, but are also associated with increased risk of cardiovascular disease (CVD) [13]. In two cohorts, the Jackson Heart Study (JHS) and the Women’s Health Initiative (WHI), the *APOL1* two-risk allele increased by two-fold the risk for myocardial infarction, stroke, and therapeutic surgical or endovascular interventions in African Americans [13]. Furthermore, *APOL1* G2 homozygous individuals were shown to be at an increased risk for stroke compared to G1 two risk alleles [13].

Although the link between hypertension and CKD is well established, CKD progression is augmented in African Americans compared to their Caucasian counterparts with similar blood pressure control [14, 15]. The *APOL1* risk variants have recently been suggested to be the missing link in the accelerated progression of hypertensive CKD despite adequate blood pressure control in African Americans [16]. In the African American Study of Kidney Disease and Hypertension comprising 693 black patients with hypertensive CKD, Parsa *et al.* [16] used a codominant genetic model to show that patients with the *APOL1* two-risk allele had a 2-fold risk of doubling their serum creatinine from baseline or developing incident end-stage renal disease over a 9-year period of follow-up. Furthermore, the progression of CKD in these patients was independent of blood pressure control [16]. The mechanism by which *APOL1* risk variants contribute to the pathogenesis of hypertensive CKD has not been elucidated. Several possible mechanisms have been suggested including a role in lipid metabolism since *APOL1* is mainly bound to high density lipoprotein [17], and variations in *APOL1* circulating levels have been associated with its genetic variants [18, 19]. Another hypothesis relates to the localization of *APOL1* protein in kidney vascular endothelium [20, 21]. In view of these studies including ours, a functional role of *APOL1* in vasculorpathology, hypertension and kidney disease is worth exploring.

The predominance of *APOL1* variants in Africans and populations with an African ancestry is linked to a natural selection, as they protect against trypanosomal infection [5] from a species that is endemic in certain regions of Africa [22]. Another factor is the differing risk of non-diabetic kidney diseases. One example is a study conducted in an Ethiopian population without HIV-associated nephropathy, which showed an absence of the *APOL1* G1 and G2 risk alleles [4, 23]. In comparison with other populations from Africa, the frequencies of *APOL1* risk alleles are relatively similar [4, 8] except those in Western African populations [4, 9–11]. Since the Cape region of South Africa is far south of the tsetse fly belt, the moderate frequency of the *APOL1* risk alleles is likely due to the African ancestry reported in this population [24].

There are some limitations to be accounted for in the interpretation of our findings. These include a low number of participants with advanced stage CKD, in spite of our large sample, which may have resulted in a reduced statistical power to uncover significant associations. Although other studies from Africa did include patients with advanced CKD, and were consequently adequately powered to capture significant effects of genes on the disease risk, these studies have however used small sample sizes to provide reliable estimates of the frequencies of those gene variants in the general population as we have done. Our study comprised participants with CKD of various etiologies that could have been either diabetic or non-diabetic. Findings could therefore be different if the study was based on a population with a more homogenous type of CKD. Our study was also limited by the heterogeneous nature of the study population, which is of mixed genetic origin with contributions from Europeans, South Asians, Indonesians and a population genetically close to the isiXhosa sub-Saharan Bantu [24], necessitating the use of ancestry informative markers to account for population stratification. Potential population stratification in an unrelated sample is known to cause spurious positive or negative associations in population-based association studies if not accounted for. However, due to financial constraints, this analysis was not conducted, raising the possibility that population admixture interfered with the association analysis. The renal disease markers were based on a single measurement in a cross-sectional study design. The cross-sectional nature of our study precludes drawing causal inferences on the direction of the associations.

## Conclusion

In conclusion, our study provides evidence that genetic variants in *APOL1* are present in a mixed-ancestry South African population, but their association with renal diseases needs further exploration in patients with non-diabetic kidney diseases.

## Material and methods

### Study participants and procedures

This investigation is based on the Bellville South cohort from Cape town, South Africa that has received study approval from the Research Ethics Committee of the Cape Peninsula University of Technology, Faculty of Health and Wellness Sciences (Reference Number: CPUT/HW-REC 2008/002 and CPUT/HW-REC 2010). The study was conducted according to the Code of Ethics of the World Medical Association (Declaration of Helsinki) [25]. All participants signed written informed consent after all the procedures had been fully explained in the language of their choice. Of the 946 self-reported mixed ancestry participants who took part in the survey, 941 consented for genetic studies. Among the latter, 72 were excluded for missing data on the genetic or renal trait variables. Therefore, 859 had valid data for the current analyses. All participants received a standardized interview and physical examination during which blood pressure was measured according to the World Health Organisation (WHO) guidelines [26] using a semi-automated digital blood pressure monitor (Rossmax PA, USA) on the right arm in a sitting position. Anthropometric measurements were performed three times and their average used for analysis: weight (kg), height (cm), waist (cm) and hip (cm) circumferences. Participants with no history of doctor-diagnosed diabetes mellitus underwent a 75 g oral glucose tolerance test (OGTT) as recommended by the WHO [27]. Further, the following biochemical parameters were determined on the Cobas 6000 Clinical Chemistry instrument (Roche Diagnostics, Germany): fasting plasma glucose, insulin, creatinine, total cholesterol (TC), high density lipoprotein cholesterol (HDL-c), triglycerides (TG), C-reactive protein (CRP), γ-glutamyltransferase (GGT), and glycated haemoglobin (HbA1c) certified by National Glycohaemoglobin Standardisation Programme (NGSP). Low density lipoprotein cholesterol (LDL-c) was calculated using Friedewald’s formula [28]. Urine albumin was determined by the immunoturbidimetric assay (Cobas 6000, Roche Diagnostics, Germany).

### SNP genotyping

Genomic DNA was extracted from whole blood samples collected in an EDTA tube. The *APOL1* single nucleotide polymorphisms (SNPs) termed G1 (rs73885319; rs60919145) and G2 (rs71785313) were genotyped using high throughput real-time polymerase chain reaction (RT-PCR) on the BioRad Optica (BioRad, USA) platform using Taqman genotyping assay (Applied Biosystems, USA). Conventional polymerase chain reaction followed by direct DNA sequencing was performed for analytical validation of high throughput genotyping.

### Definitions and calculations

Body mass index (BMI) was calculated as weight per square meter (kg/m2) and waist-hip-ratio (WHR) as waist/hip circumferences (cm). Type 2 diabetes status was based on a history of doctor-diagnosis, a fasting plasma glucose ≥7.0 mmol/l and/or a 2-hour post-OGTT plasma glucose ≥11.1 mmol/l. Hypertension was based on a history of doctor diagnosed hypertension and/or receiving medications for hypertension or average systolic blood pressure ≥140 mmHg and/or average diastolic blood pressure ≥90 mmHg. Urinary albumin excretion was quantified in term of urinary albumin/creatinine ratio (ACR). Glomerular filtration rate (GFR) was estimated by the 4-variable Modification of Diet in Renal Disease (MDRD) equation applicable to standardised serum creatinine values [29, 30], and the Chronic Kidney Disease Epidemiology Collaboration (CKD-EPI) equation [31].

### Statistical analysis

General characteristics of the study group are summarized as count and percentage for dichotomous traits, mean and standard deviation (SD) or median and 25^th^-75^th^ percentiles for quantitative traits. Traits were log-transformed to approximate normality, where necessary, prior to analysis. SNPs were tested for departure from Hardy-Weinberg Equilibrium (HWE) expectation via a chi square goodness of fit test. Linkage disequilibrium (LD) was estimated using the D’ statistic. Linear regression models were used for the analysis of quantitative traits and logistic regression models for dichotomous traits, assuming both dominant and log-additive genetic models for the SNPs. Using linear and logistic models enabled us to adjust all analyses for known confounders as specified everywhere in the results. We investigated the association of each SNP with each trait, overall and tested for heterogeneity by major subgroups by adding the interaction term of major grouping variables and each SNP to a model that contained the main effects of grouping variable and the relevant SNP. Results corresponding to p-values below 5 % are described as significant. Adjustment for multiple testing was conducted via Bonferroni methods. All analyses used the R statistical software (version 3.0.3 [2014-03-06], The R Foundation for statistical computing, Vienna, Austria) and the packages ‘*genetics*’ and ‘*SNPassoc*’.
